# Non-A non-B acute aortic dissection with entry tear in the aortic arch

**DOI:** 10.1093/icvts/ivab375

**Published:** 2022-02-07

**Authors:** Monika Kosiorowska, Mikolaj Berezowski, Kazimierz Widenka, Maximilian Kreibich, Friedhelm Beyersdorf, Martin Czerny, Bartosz Rylski

**Affiliations:** 1 Department of Cardiovascular Surgery, Faculty of Medicine, Heart Centre, University of Freiburg, Freiburg, Germany; 2 Faculty of Medicine, Albert-Ludwigs-University, Freiburg, Germany; 3 Department and Clinic of Cardiac Surgery, Wroclaw Medical University, Wroclaw, Poland; 4 Department of Cardiac Surgery, University Hospital No 2, Rzeszow, Poland

**Keywords:** Non-A non-B aortic dissection, Thoracic endovascular aortic repair, Frozen elephant trunk

## Abstract

**OBJECTIVES:**

Our aim was to describe the outcomes of the latest treatment options of acute non-A non-B aortic dissection involving an entry tear in the aortic arch.

**METHODS:**

Included were patients who presented between January 2001 and February 2020 with a non-A non-B aortic dissection involving the aortic arch but not the ascending aorta and with the most proximal entry tear located within the aortic arch between the innominate and left subclavian artery. Clinical data and operative details were retrieved from medical histories and surgical protocols. Preoperative, postoperative and follow-up computed tomography angiography scans were analysed.

**RESULTS:**

We analysed a total of 39 patients [median age 62 (52; 67) years, men 76.9%] with non-A non-B arch entry aortic dissections type. They underwent 15 thoracic endovascular aortic repairs, 20 frozen elephant trunk implantations, 1 hybrid arch replacement, or 1 conventional arch replacement. Two patients were managed conservatively. Twelve (31%) patients underwent emergent intervention, 12 (31%) were treated invasively within 2 weeks. Another 2 (5%) and 9 (23%) patients were treated 2 and 4 weeks after dissection occurred, respectively. Six (15%) patients presented with an impending aortic rupture, while 19 (49%) had at least one malperfused organ. Four patients (27%) died after thoracic endovascular aortic repair; the 30-day mortality following frozen elephant trunk was 0%.

**CONCLUSIONS:**

Non-A non-B acute aortic dissection reveals a frequently complicated course requiring emergency intervention. The majority of patients required aortic arch repair within the first 2 weeks. Total arch replacement with the frozen elephant trunk technique seems to be low procedural mortality, and may become the treatment of choice in arch entry non-A non-B aortic dissection.

**Figure:**
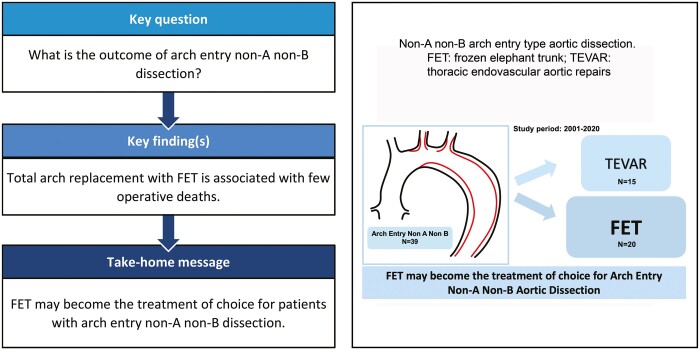

## INTRODUCTION

Acute aortic dissection’s current management depends on the dissection’s anatomy including the entry tear location and dissection extension, and on the malperfusion status [1]. Aortic arch dissection in patients with a non-dissected ascending aorta has recently been described as non-A non-B dissection (type, entry site, malperfusion classification: TEM non-A non-B) [1, 2]. Two types of non-A non-B aortic dissection have been defined: descending entry and arch entry type [2]. In the descending entry type, the entry tear is located distal to the left subclavian artery (LSA), and there is a retrograde extension of the dissection into the aortic arch, while in the arch entry type the entry tear is located between the innominate and left subclavian arteries. To date, there is no consensus on what constitutes the best management for these aortic anomalies [3]. While the descending entry type non-A non-B aortic dissection frequently reveals a course resembling that of the type B aortic dissection, arch entry type dissections often become complex and require emergency intervention. Recent reports suggest that both thoracic endovascular aortic repair (TEVAR) and conservative therapy are associated with worse survival and a high rate of aortic complications in these patients, while open aortic arch repair seems to yield better outcomes [4].

Our purpose was to describe the outcomes of currently available treatment options in patients presenting an acute non-A non-B aortic dissection with entry tear in the aortic arch.

## MATERIALS AND METHODS

### Ethical statement

Our institutional review committee approved this study and the need for informed consent was waived (Ethics Committee Freiburg University, approval number 289/14).

### Study population

Our aortic dissection database was searched for all patients with an acute aortic dissection admitted to the Heart Centre Freiburg University between January 2001 and February 2020. Patients were included in this retrospective observational study who presented with a non-A non-B aortic dissection (i.e. dissection involving the aortic arch but not the ascending aorta) and with the most proximal entry tear located within the aortic arch (between the innominate and LSA). Non-A non-B dissection patients with the entry tear located distal to the LSA were excluded. The reporting in this study based on STORAGE guidelines [3].

### Image analysis

Aortic diameters, dissection extension and the most proximal entry tear location were analysed according to electrocardiography gated computed tomography angiography (CTA). Analyses were performed using Impax EE (Agfa HealthCare N.V., Morstel, Belgium). All the measurements were taken in multiplanar reconstruction always in a plane perpendicular to the manually corrected local aortic centreline. Maximal total aortic and maximal true lumen diameters, and minimal diameters were measured at the level of the mid-ascending aorta, aortic arch between the left common carotid artery and LSA, descending aorta (at maximal diameter) and abdominal aorta (at maximal diameter). CTAs obtained at admission, CTAs after aortic repair, CTA before reintervention and CTAs at the last follow-up were analysed.

### Patient management

The anatomy of the aortic dissection and organ perfusion was carefully assessed in all patients radiologically and clinically at admission. Patients presenting initially with hypertension or pain only, but without malperfusion or aortic rupture, were managed conservatively. Patients with end-organ malperfusion defined by clinical (pulseless and cold extremities, severe abdominal pain), laboratory (elevated serum lactate) and imaging evidence (collapsed true aortic lumen, dissected visceral arteries with significantly narrowed true lumen by a thrombosed false lumen) or aortic rupture were treated endovascularly or in open fashion on an emergency basis. Endovascular treatment involved TEVAR entailing carotid-subclavian bypass (Zone 2) or transposition of both the left carotid and left subclavian arteries (TEVAR Zone 1) as well as isolated stenting of dissected visceral vessels. The hybrid approach included sternotomy for supra-aortic vessels debranching with Dacron bypasses anastomosed on the ascending aorta and TEVAR with the stent graft landing in the distal ascending aorta (TEVAR Zone 0). Open surgery included aortic arch replacement usually with the frozen elephant trunk (FET) technique or malperfused organ revascularization with bypasses. The decision on an endovascular, hybrid or open approach was made individually by the surgeon.

Patients exhibiting no organ malperfusion, no aneurysm and no aortic rupture were treated similarly to the Type B dissection patients and admitted to the intensive care unit for blood pressure monitoring, anti-hypertensive therapy and analgesia with interval radiographic evaluation of their aortic anatomy.

Because of the growing evidence of the benefits of FET surgery in acute aortic dissection patients since January 2018 [5, 6], all non-A non-B arch entry type aortic dissection patients underwent total aortic arch replacement with FET on an emergent or urgent basis.

Definitions: arch configurations: (Type I—all 3 great vessels originate in the same horizontal plane as the outer curvature of the aortic arch, Type II—the IA originates between the horizontal planes of the outer and inner curvatures of the aortic arch, Type III—the IA originates below the horizontal plane of the inner curvature of the aortic arch); primary endpoint is the early- and long-term mortality; secondary endpoint is the reintervention rate.

### Statistical analysis

Continuous data are reported as median (first quartile; third quartile), while categorical variables are shown as counts, percentages. The cumulative survival curve for long-term follow-up was constructed using the Kaplan–Meier method. Due to such low patient numbers, no statistical comparison between subgroups defined by treatment modality was made. SigmaPlot 12.3 (Systat Software, San Jose, CA, USA) was used for the Kaplan–Meier analysis.

## RESULTS

**Figure 1:**
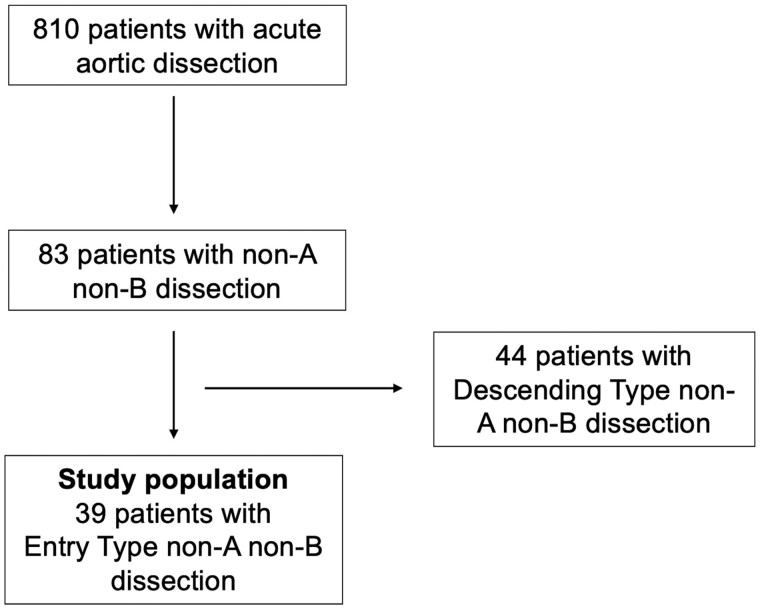
Flowchart of patient selection.

Overall, 39 patients [median age 62 (first quartile 52; third quartile 67) years, men 76.9%] were included in this study (Fig. 1). Patients were followed up during the past 19 years in our aortic outpatient clinic at a 1-year interval. Median follow-up was 2.6 (first quartile 1.5; third quartile 4.7) years, whereas the calculated follow-up index was 0.7. Six (15%) patients were admitted displaying signs of impending aortic rupture, 2 of whom were in cardiogenic shock. Malperfusion of at least one organ at the admission was observed in 19 (49%) patients (Table 1).

**Table 1:** Cardiovascular risk profile and clinical presentation

Parameters	*N* = 39
Demographics	
Age, years	62 (52; 67)
Male gender	30 (76.9)
Risk factors	
Hypertension	37 (94.9)
Dyslipidaemia	11 (28.2)
Diabetes	3 (7.7)
Smoking history	12 (30.8)
Renal failure	3 (7.7)
Coronary artery disease	5 (12.8)
COPD	5 (12.8)
Peripheral artery disease	1 (2.6)
BAV	3 (7.7)
Marfan syndrome	4 (10.3)
Obesity	7 (17.9)
Previous cardiac surgery	10 (25.6)
Dissection aetiology	
Spontaneous	37 (94.9)
Iatrogenic	2 (5.1)
Clinical presentation at admission	
Cardiogenic shock	2 (5.1)
Tamponade	2 (5.1)
Cardiac arrest	1 (2.6)
Syncope	2 (5.1)
Aortic rupture	6 (15.4)
Malperfusion	
At least 1 organ	19 (48.7)
Gastrointestinal	9 (23.1)
Renal	8 (20.5)
Iliofemoral	6 (15.4)
Cerebral	2 (5.1)
Spinal	1 (2.6)
Persistent pain	26 (66.7)
Refractory hypertension	12 (30.8)

Categorical variables are indicated as counts and percentages, continuous as median (first quartile; third quartile).

BAV: bicuspid aortic valve; COPD: chronic obstructive pulmonary disease.

### Aortic anatomy

Type I, II and III aortic arch configurations were observed in 28%, 62% and 10% of patients, respectively. Aortic arch dissection extending proximally up to the left common carotid artery orifice was diagnosed in 30 (77%), and up to the innominate artery in 9 (23%) patients. Eight (21%) patients had the most proximal entry tear located in the proximal part of the aortic arch between the innominate artery and left common carotid artery. In all but 1 patient, the entry was located within the arch convexity. Maximal aortic diameters measured at the initial CTA were 42 mm (37; 45.0), 36 mm (34; 42), 39 mm (35; 42) and 31 mm (27; 33) at the ascending aorta, aortic arch, descending and abdominal aorta, respectively (Table 2).

**Table 2:** Aortic anatomy after non-A non-B arch entry type dissection onset

Parameters	*N* = 39
Aortic arch configuration	
Type I	11 (28.2)
Type II	24 (61.5)
Type III	4 (10.3)
Dissection proximal extension up to	
Innominate artery	9 (23.1)
LCCA	30 (76.9)
Aortic diameter	
Ascending aorta	42 (36.9; 45.0)
Aortic arch	36 (34.2; 42.1)
Descending thoracic aorta	39 (35.2; 42.0)
Abdominal aorta	31 (27.2; 33.2)
Entry location	
Arch convexity	38 (97.4)
Arch concavity	1 (2.6)

Categorical variables are given as counts and percentages, continuous as median (first quartile; third quartile). Diameters are indicated in mm.

LCCA: left common carotid artery.

### Approach

To date, 20 patients have undergone FET, 15 TEVAR, one a conventional arch replacement and another underwent a 1 hybrid approach including ascending-innominate and ascending-left carotid bypass and TEVAR Zone 0 (LSA was ligated and not revascularized). The patient primarily treated via hybrid arch repair died 2 days after his second reintervention (TEVAR with distal extension for large aortic aneurysm caused by aortic rupture). The patient who underwent conventional arch replacement required TEVAR 5 years after his primary surgery. Two patients are still being treated conservatively as they have both refused surgery.

The first FET procedure in a patient with arch entry type non-A non-B aortic dissection was in 2010, however, more frequent application of this treatment started in 2016. Since January 2018, the FET has become a first-line treatment and has been applied in all non-A non-B arch entry type aortic dissection patients admitted to our centre.

### Timing

Of the 39 patients, 12 (31%) who required emergent intervention, 5 underwent TEVAR, 6 total arch replacements with FET and 1 visceral vessel stenting.

Additionally, 2 patients became haemodynamically unstable during their hospital stay one and 8 days after admission, respectively, and required an emergency intervention: one because of aortic rupture, another due to a progressing dissection proximal to the supra-aortic vessels and distal to iliac arteries. Data on the indications and timing of interventions are summarized in Table 3.

**Table 3:** Timing and indication for intervention

Parameter	FET (*n* = 20)	TEVAR (*n* = 15)
Emergency after admission	6 (30.0)	5 (33.3)
Aortic rupture	2 (10.0)	3 (20.0)
Multiorgan malperfusion^a^	1 (5.0)	0
Visceral and iliofemoral malperfusion	0	2 (13.3)
Visceral malperfusion	1 (5.0)	0
Renal malperfusion	1 (5.0)	0
Suspected Type A aortic dissection	1 (5.0)	0
Emergency during hospital stay	1 (5.0)	1 (6.7)
Aortic rupture	0	1 (6.7)
Dissection progression	1 (5.0)	0
Urgent (<2 weeks)	7 (35.0)	4 (26.7)
Persistent pain	2 (10.0)	1 (6.7)
Large aortic aneurysm	3 (15.0)	0
Rapid aortic growth	1 (5.0)	1 (6.7)
Iliofemoral malperfusion	0	1 (6.7)
Dissection progression	1 (5.0)	0
Refractory hypertension	0	1 (6.7)
Elective (>2 weeks)	1 (5.0)	1 (6.7)
Large aortic aneurysm	1 (5.0)	0
Rapid aortic growth	0	1 (6.7)
At follow-up (>4 weeks)	4 (20.0)	4 (26.7)
Large aortic aneurysm	4 (20.0)	2 (13.3)
Persistent pain	0	1 (6.7)

Categorical variables are indicated as counts and percentages, continuous as median (first quartile; third quartile).

a Multiorgan malperfusion, abnormal perfusion involving 2 or more organ systems.

FET: frozen elephant trunk; TEVAR: thoracic endovascular aortic repair.

### Thoracic endovascular aortic repair

Overall, TEVAR was performed in 15 (39%) patients; in 5 of them on an emergency basis. The most proximal entry tear was closed in 7 (47%) patients. Four patients whose intervention failed to close the entry tear required an additional aortic intervention for thoracic aortic aneurysm, and 1 for retrograde Type A dissection. One patient is still being treated conservatively. Two patients died at 3 weeks and 1.5 years after TEVAR, both for unknown reasons.

In total, 10 (67%) patients required secondary aortic intervention. Four of them as a consequence of the unsuccessful entry tear closure. Subsequent 3 underwent ascending aortic replacement due to retrograde Type A aortic dissection. Two patients required a reintervention because of endoleak Type Ia and Ib treated with TEVAR extension. One patient underwent a femoro-femoral crossover bypass implantation for lower limb ischaemia, and a coil embolization for a type II endoleak. We noted 1 carotid-subclavian bypass for left upper limb ischaemia. One patient required aortic valve replacement due to a (dissection un-related) severe aortic stenosis within 6 months after TEVAR. Two patients required a thoracotomy to stem postoperative bleeding.

Overall, 4 (27%) patients died within 30 days after TEVAR. Two patients died in the operating theatre after the emergency repair of an aortic rupture due to haemorrhagic shock. One patient who had undergone emergency surgery for aortic rupture died during their stay in hospital from stroke, and the other one died 16 days after an emergency intervention from a reason unknown. TEVAR results are summarized in Table 4.

**Table 4:** Treatment details

Parameter	FET (*N* = 20)	TEVAR (*N* = 15)
Proximal landing zone		
Zone 1	N/A	2 (13.3)
Zone 2	N/A	10 (66.7)
Zone 3	N/A	3 (20.0)
Surgery timing		
Emergency	6 (30.0)	5 (33.3)
Urgent (<2 weeks)	7 (35.0)	4 (26.7)
Elective (>2 weeks)	1 (5.0)	1 (6.7)
At follow-up (>4 weeks)	5 (25.0)	4 (26.7)
Emergency after cardiac decompensation during in-hospital stay	1 (5.0)	1 (6.7)
Concomitant procedure		
Aortic valve replacement	4 (20.0)	N/A
Coronary artery bypass grafting	2 (10.0)	N/A
Tricuspid valve repair	1 (5.0)	N/A
Other	3 (15.0)	N/A
Outcome		
Retrograde type A dissection	0	3 (20.0)
Circulatory failure	0	2 (13.3)
Respiratory failure	1 (5.0)	1 (6.7)
Cerebrovascular event	1 (5.0)	0
Exploration for bleeding	0	2 (13.3)
Lower limb ischaemia	0	2 (13.3)
Renal failure	1 (5.0)	0
Endocarditis	1 (5.0)	0
Endoleak		
Ia	N/A	1 (6.7)
Ib	0	1 (6.7)
II	0	2 (13.3)
D-SINE	1 (5.0)	0
Adverse aortic events	7 (35.0)	11 (77.3)
ICU stay (days)	4 (3; 5.75)	2 (1; 3)
In-hospital stay (days)	19 (15; 24.5)	13 (9; 41.5)
In-hospital mortality	0	4 (26.7)
Aortic reinterventions		
Overall	7 (35.0)	10 (66.7)
Distal aortic reinterventions	6 (30.0)	3 (20.0)
Open surgery	1 (5.0)	6 (40.0)
More than 1 aortic reintervention	0	1 (6.7)

Categorical variables are indicated as counts and percentages, continuous as median (first quartile; third quartile).

D-SINE: distal stent graft-induced new entry; FET: frozen elephant trunk; ICU: intensive care unit; TEVAR: thoracic endovascular aortic repair.

### Total arch replacement with frozen elephant trunk

A total of 20 (56%) patients underwent total arch replacement with FET technique. Surgery took place in an emergency setting on the admission day in 6 patients; one became unstable during in-hospital stay and required an emergency intervention 1 day after their dissection onset.

Among 8 patients who suffered complications after the primary repair, we noted: 1 cerebrovascular event, 1 respiratory failure, 1 renal failure, 1 endocarditis and 1 distal stent graft-induced new entry. Seven (35%) patients required an aortic reintervention. Distal extension with TEVAR was performed due to a worsening descending aortic aneurysm in 6, and a distal stent graft-induced new entry in 1 patient. Another patient received a mitral valve replacement due to endocarditis. There was no mortality among FET-treated patients FET outcomes are listed in Table 4.

### Overall outcomes

To sum up our results: 5 of 39 (13%) non-A non-B arch entry type patients died perioperatively. In-hospital mortality resulted from aortic rupture in 3 patients. Median hospital time was 18 (11; 25) days. Seventeen (44%) patients required a secondary aortic intervention. Supplementary Material illustrates the freedom from adverse aortic events.

## DISCUSSION

Non-A non-B aortic dissection involving an entry tear in the aortic arch is fortunately an infrequent condition, and there is little evidence on its outcome. The course of this type of aortic dissection often seems to be more complicated than that of a Type B or Type non-A non-B dissection with an entry tear in the descending aorta [7, 8]. In light of the growing evidence on managing acute descending dissection, and the long-term benefit from closing the most proximal entry tear (leading to positive aortic remodelling), more and more surgeons are now deciding to intervene earlier [9, 10].

We previously reported our centre’s experience on the treatment of non-A non-B aortic dissection with the entry tear in both the aortic arch and descending aorta [2]. We found that patients with acute non-A non-B aortic dissection often required emergency aortic repair at admission due to organ malperfusion or aortic rupture. Aortic interventions were necessary in most non-A non-B dissection patients within 2 weeks after their dissection onset.

In this article, we have aimed to describe our results from a larger patient sample including up-to-date follow-ups focusing on the non-A non-B aortic dissection with an entry tear in the aortic arch.

Our study findings can be summarized as follows:

- Acute non-A non-B arch entry type dissection patients frequently require an emergent intervention because of their clinical state at admission, which is usually exacerbated by an aortic rupture or end-organ malperfusion.
- TEVAR is associated with high in-hospital mortality, and TEVAR patients frequently needed a reintervention at follow-up.
- TEVAR did not always result in the closure of the most proximal entry tear—leading to a worse clinical outcome
- Total arch replacement with FET is associated with low procedural mortality and no risk of retrograde aortic dissection type A.

### Relationship to previous studies

A few reports on non-A non-B aortic dissection were recently published: a meta-analysis by Carino *et al.* [4] summarized knowledge extracted from the literature on arch dissection not involving the ascending aorta. The lesson taken from their very recent review is—in line with our findings—that the overwhelming majority of patients with non-A non-B aortic dissections had a complicated course requiring intervention. They also found that the 30-day mortality of patients who underwent medical therapy was 14%—substantially higher than that associated with type B dissections. Considering their evidence and the very low after—FET mortality observed in our series, we find that an invasive, early intervention seems to be a reasonable option in arch entry non-A non-B dissection patients.

### Classification controversies

The paucity of guidelines on non-A non-B aortic dissection is not only the result of its low incidence (2–5.5% among all dissections [7, 10–12]). Another reason might be that its pathology is not covered in either the Stanford or DeBakey classifications. A new classification system based on the Type of dissection, Entry location and Malperfusion status (TEM) including non-A non-B type was described last year [1]. We believe that TEM has the potential to improve the decision-making process including a rapid assessment of the patient’s prognosis together with the choice and timing of the intervention. The universality of its universal application considers the dissection’s specific anatomy and the entry tear’s location, as well as the patient’s clinical state—all factors that were not previously addressed. Considering the rapidity and severity of aortic dissection, demanding on-the-spot decisions and clear communication pathways, this new classification gives surgeons an excellent tool to facilitate patient management, and could potentially even improve survival.

### Open aortic arch replacement

FET technique comprises open arch surgery and TEVAR. It is a sound treatment option for many aortic arch pathologies. A stable proximal landing zone created through FET facilitates later distal interventions. Another advantage is the elimination of proximal complications such as retrograde Type A dissection (a notorious problem after TEVAR) [4, 13]. Our FET patients revealed no in-hospital mortality. Another FET advantage is that it enables surgeons to replace a dilated ascending aorta or to execute other necessary heart surgery concomitant to the FET procedure. We therefore maintain that total arch replacement with FET technique is an effective and reliable treatment option for acute arch entry non-A non-B dissection patients.

### Hybrid repair

Recently, Wang *et al.* [14] introduced hybrid treatment (inclusion aortic arch technique) for 28 patients with non-A non-B aortic dissection and an entry tear in the aortic arch as a safe and promising treatment modality. They surgically closed the arch entry tear through a longitudinal aortic arch incision and fixed the trimmed proximal part of the FET prosthesis with a Prolene suture to the aortic arch around the cerebral vessels. This enabled a faster procedure and less time in hypothermic circulatory arrest, since they did not need to replace the aortic arch vessels. Although their short-term results have been encouraging, caution is needed about the fate of non-replaced parts of the dissected aortic arch. In our series, 1 patient underwent hybrid repair. Two days after his second reintervention (a TEVAR distal extension for large aortic aneurysm), he died from an aortic rupture.

### Thoracic endovascular aortic repair

TEVAR is claimed to be a good option for a descending entry type non-A non-B aortic dissection [4]. In proximal entry non-A non-B aortic dissection patients, the non-dissected aorta, which might serve as a proximal landing zone, is in arch zone 0–1. TEVAR zone 0–1 is associated with a higher incidence of retrograde type A aortic dissection (20% in our series), a condition requiring emergency conversion to open surgery. Moreover, TEVAR in arch entry dissections is associated with a high rate of proximal complications at follow-up. TEVAR in many patients failed to close the entry tear in the aortic arch, resulting in significantly worse clinical course. Furthermore, this group’s 30-day mortality was 27%. It seems that TEVAR should be carried out with great caution and if so, only in specific, well-selected patients.

### Study limitations

This is a retrospective, single-centre study relying on a relatively low number of patients insufficient for adequate subgroup analysis and reducing the effects of confounding. Our data were collected over a long time period lasting 20 years, and selection bias cannot be excluded. Moreover, the patients distribution imbalance over the years and the evolving endovascular and surgical treatment might have affected the results. The decision on an endovascular, hybrid or open approach was made individually by the surgeon. The location of entries observed in our study was investigated via CTA, thus not all the entries in an acutely dissected aorta are demonstrated.

## CONCLUSIONS

The presentation of arch entry non-A non-B acute aortic dissection is frequently complicated, and it requires an early intervention on an emergency basis—at admission or shortly thereafter. The majority of our study patients needed aortic arch repair within the first 2 weeks. As total arch replacement with FET technique seems to be associated with low procedural mortality, it may become the treatment of choice for arch entry non-A non-B aortic dissections.

## SUPPLEMENTARY MATERIAL

Supplementary material is available at *ICVTS* online.

## Funding

None.

**Conflict of interest:** Martin Czerny is a consultant to Terumo Aortic and Medtronic, received speaking honoraria from Bentley and Cryolife, and is a shareholder of TEVAR Ltd. Bartosz Rylski is a consultant to Terumo Aortic.

### Data Availability Statement

The data underlying this article will be shared on reasonable request to the corresponding author.

### Author contributions

**Monika Kosiorowska:** Data curation; Investigation; Resources; Writing—original draft. **Mikolaj Berezowski:** Project administration; Resources; Writing—original draft. **Kazimierz Widenka:** Supervision; Writing—review & editing. **Maximilian Kreibich:** Supervision; Writing—review & editing. **Friedhelm Beyersdorf:** Supervision; Writing—review & editing. **Martin Czerny:** Supervision; Writing—review & editing. **Bartosz Rylski:** Conceptualization; Formal analysis; Project administration; Supervision; Validation; Writing—review & editing.

### Reviewer information

Interactive CardioVascular and Thoracic Surgery thanks Gabriele Piffaretti, Eugenio Neri, Yukitoshi Shirakawa and the other anonymous reviewer(s) for their contribution to the peer review process of this article.

## Supplementary Material

ivab375_Supplementary_Data

## Notes

Presented at the 34rd Annual Meeting of the European Association of the Cardio-Thoracic Surgery, Barcelona, Spain, 8–10 October 2020.

## ABBREVIATIONS

CTA: Computed tomography angiography
FET: Frozen elephant trunk
TEVAR: Thoracic endovascular aortic repair
