# Supplementary figures and images for: Non-A non-B acute aortic dissection with entry tear in the aortic arch
Source: Interact Cardiovasc Thorac Surg. 2022 Feb 7;34(5):878–84. doi: 10.1093/icvts/ivab375 (PMC9070470; doi:10.1093/icvts/ivab375)

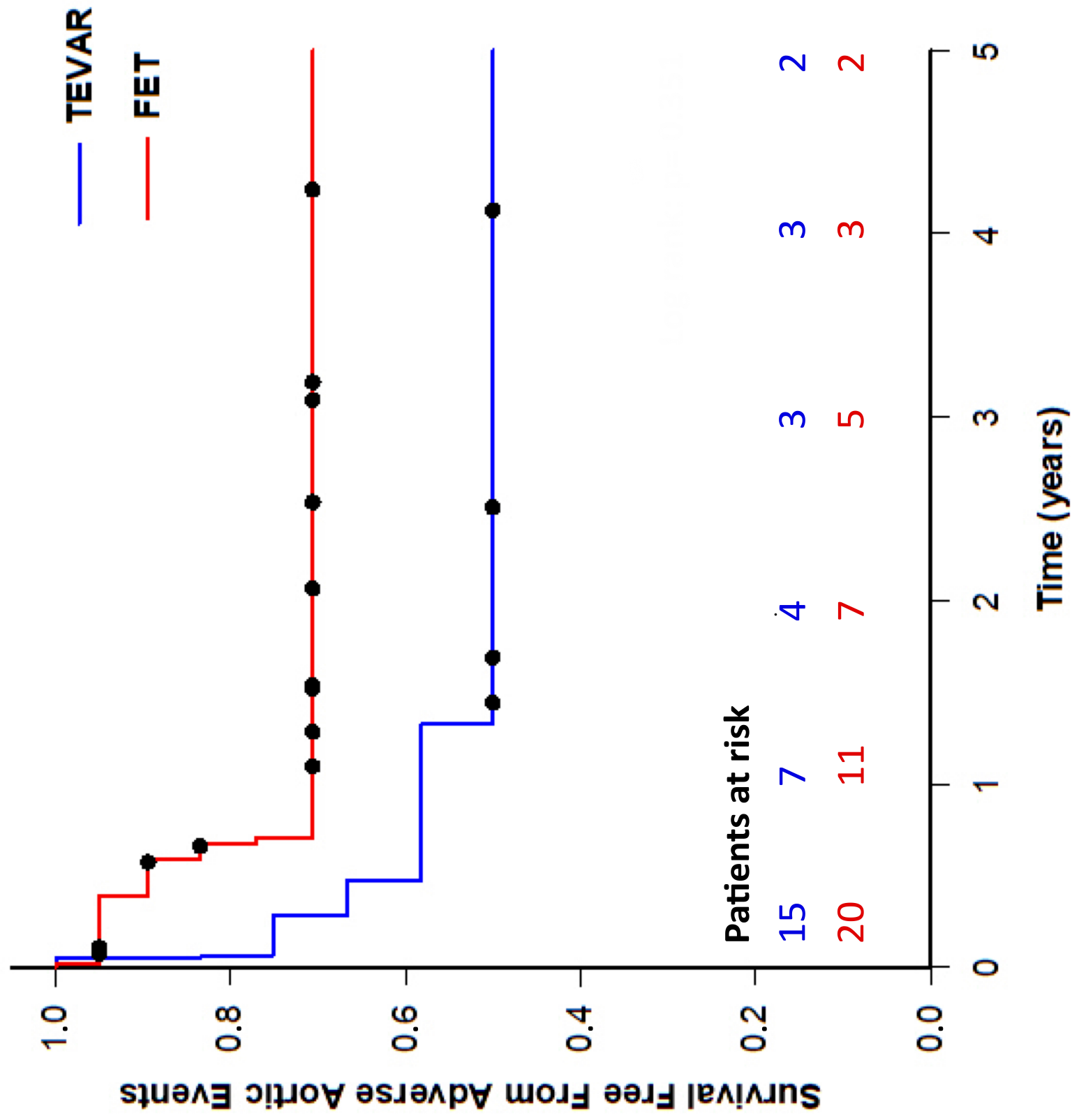

Supplement: ivab375_Supplementary_Data [file ivab375_supplementary_data.pdf]
